# Associations of Prenatal Mercury Exposure From Maternal Fish Consumption and Polyunsaturated Fatty Acids With Child Neurodevelopment: A Prospective Cohort Study in Italy

**DOI:** 10.2188/jea.JE20120168

**Published:** 2013-09-05

**Authors:** Francesca Valent, Marika Mariuz, Maura Bin, D’Anna Little, Darja Mazej, Veronica Tognin, Janja Tratnik, Alison J McAfee, Maria S Mulhern, Maria Parpinel, Marco Carrozzi, Milena Horvat, Giorgio Tamburlini, Fabio Barbone

**Affiliations:** 1 Institute of Hygiene and Clinical Epidemiology, University Hospital of Udine, Udine, Italy; 2 Institute of Hygiene and Epidemiology, Department of Medical and Biological Sciences, University of Udine, Udine, Italy; 3 Division of Child Neuropsychiatry, Institute for Maternal and Child Health-IRCCS Burlo Garofolo, Trieste, Italy; 4 Jozef Stefan Institute, Ljubljana, Slovenia; 5 School of Biomedical Sciences, University of Ulster, Coleraine, UK; 6 Institute for Maternal and Child Health-IRCCS Burlo Garofolo, Trieste, Italy; 7 International Postgraduate School Jožef Stefan, Ljubljana, Slovenia

**Keywords:** mercury, polyunsaturated fatty acids, nervous system development, fish, Bayley Scales of Infant and Toddler Development

## Abstract

**Background:**

Mercury is a neurotoxin, and limited prenatal exposure to it can affect long-term child neurodevelopment. However, results of epidemiologic studies of such exposure have been inconsistent. We examined the association of prenatal mercury exposure from maternal fish consumption with child neurodevelopment in northern Italy.

**Methods:**

A population-based cohort of 606 children and their mothers was studied from pregnancy to age 18 months. Mercury levels were measured in maternal hair and blood during pregnancy and in umbilical cord blood and breast milk. Levels of polyunsaturated fatty acids (PUFAs) were measured in maternal serum. Maternal and child intakes of fish were assessed by using a food frequency questionnaire. The Bayley Scales of Infant and Toddler Development, Third Edition (BSID-III) was used to evaluate child neurodevelopment. Multivariate linear regression was used to examine the association of mercury exposure with BSID-III scores, after controlling for maternal fish intake, PUFAs during pregnancy, and several other confounders.

**Results:**

Mean weekly fish intake during pregnancy was less than 2 servings. Mercury concentrations in biological samples were low (mean, 1061 ng/g in hair) and moderately correlated with fish intake, particularly of carnivorous species. Maternal ω-3 PUFA concentrations were poorly correlated with fish intake. Maternal intelligence quotient (IQ) and child intake of fish were significantly associated with neurodevelopment scores. In multivariate models, the level of Hg exposure was not associated with neurodevelopmental performance at 18 months.

**Conclusions:**

In this Italian population, neurodevelopment at 18 months was associated with child intake of fresh fish and maternal IQ rather than with mercury exposure. The expected beneficial effect of maternal fish intake (from maternal ω-3 PUFAs) was not found.

## INTRODUCTION

Biomethylation of inorganic mercury (Hg) in aquatic sediments produces methylmercury (MeHg), which accumulates in all fish, although concentrations are higher in larger and older predatory fish.^1^ MeHg is a neurotoxin and its toxicity, particularly to the human fetus, became evident after 2 major incidents, in Minamata^2^ and Iraq,^3^ of food contamination (seafood and bread, respectively) resulted in exposures of populations to high doses of MeHg. Since then, there have been concerns that adverse neurodevelopmental effects could occur even at relatively low doses, ie, <10 ppm (1 ppm = 1000 ng/g) of total mercury (THg) in hair. Such concentrations have been observed in populations that consume large amounts of fish.^4^

Two large prospective cohort studies have investigated the effects of prenatal Hg exposure on child neurodevelopment. One was conducted in the Faroe Islands, where a considerable amount of pilot whale meat was consumed, and found that such exposures may have detrimental effects on neurodevelopment.^5^^–^^8^ The other study was conducted in the Seychelles Islands, where the population consumes numerous fish meals per week, and found no negative associations between Hg and child development.^4^^,^^9^^–^^14^ In their careful analysis, Schoeman et al^15^ offered several reasons to explain why different results were obtained from these 2 cohorts despite their similar overall Hg exposures: the studies used different outcomes and different biological samples (umbilical cord blood vs maternal hair) to assess Hg exposure, they controlled for different potential confounders, the characteristics of the study populations differed (including genetic differences and different concentrations of Hg and other contaminants in fish and seafood), and there may have been a differential effect due to the number of meals of highly contaminated fish and whale meat.

Fish is also a source of eicosapentaenoic acid (EPA, 20:5 ω-3) and docosahexaenoic acid (DHA, 22:6 ω-3), which are long-chain polyunsaturated fatty acids (LCPUFAs) that have central roles in fetal growth and neurodevelopment. They are transferred from mother to fetus via the placenta.^16^ Results from observational studies indicate that LCPUFAs might explain the increased risk of poorer neurologic and behavioral performance among children whose mothers had lower fish intakes during pregnancy.^17^^–^^19^ Furthermore, the unexpected improvement of some outcomes among children of mothers with increased hair Hg concentrations—observed in the Seychelles Child Development Study—might be due result to higher intakes of LCPUFAs from fish.^11^ In the Seychelles Child Development Nutrition Study, maternal serum ω-3 LCPUFA concentrations during the last trimester of pregnancy were positively associated with child psychomotor development at age 9 months^20^ and 30 months.^21^

In Italy, a small cohort study (*n* = 242) showed that the mean THg concentration in the hair of pregnant women living in the coastal area of the Friuli Venezia Giulia region was low (1.33 ppm) but was as high as 8 ppm in some women, and THg concentration was directly associated with reported intake of large local predatory fish.^22^^,^^23^ The Denver Developmental Screening Test II (DDST-II) was used to screen 52 children at age 18 months, and the THg concentration in the hair of mothers of those with above–age-level fine motor-adaptive function was significantly lower than that in the hair of other women.^22^ However, the statistical power of the study was low due to the small sample size, and there was no information on potential confounding variables such as concentrations of maternal PUFAs in pregnancy and some social and parenting indicators.

We established a new larger cohort in the Mediterranean coastal area of northeastern Italy to investigate the association of prenatal Hg exposure from maternal fish consumption with child neurodevelopment, with additional consideration of maternal nutritional factors.

## METHODS

In this report, we describe the results of the Italian component of a research project that involved other Mediterranean coastal areas. The protocol of the study is described in detail elsewhere^24^ and was approved by the Ethics Committees of the University of Udine and IRCCS Burlo Garofolo Children’s Hospital of Trieste.

### Study population

Pregnant women at 20 to 22 weeks of gestation were recruited from the Burlo Garofolo Hospital, in northeast Italy, from April 2007 to April 2009. Inclusion and exclusion criteria, and a detailed description of the study protocol, have been published elsewhere.^24^ In brief, eligible women provided a small (1-gram) hair sample at the time of recruitment. They were visited again when they had their next ultrasound scan, at approximately 32 weeks of gestation. At 32 weeks, a sample of maternal urine was collected. A blood sample was collected during weeks 20 to 22 (whenever possible) or during week 32 (if the woman refused blood withdrawal during week 20 to 22 due to lack of time or other personal reasons). During pregnancy, the women underwent a test of general intelligence. At delivery, a sample of cord blood was collected, and a questionnaire was given to them, to be completed during the following month. The women were visited again at their homes, approximately 1 month after delivery, and the questionnaire was collected. The children underwent neurodevelopmental evaluation at the Burlo Garofolo Hospital at approximately 18 months of age. On the same occasion, their parents completed a supplementary questionnaire, to update information on the family and child.

### Exposure variables

THg and MeHg concentrations were measured in women’s hair collected at recruitment, and in cord blood.

A large cohort study of pregnant women in the United Kingdom found that fish consumption changed little during pregnancy.^25^ Therefore, we assessed average maternal fish intake throughout pregnancy by using the 138-item food frequency section of the detailed semi-structured questionnaire that was completed after delivery. The section was adapted from a validated food frequency questionnaire.^26^^,^^27^ In addition, to identify the fish species preferred by pregnant women in the study population, a special subsection of the food frequency module was used for semiqualitative assessment of consumption of 22 fish species commonly fished or marketed in the study area.

### Outcome

Neurodevelopmental testing at age 18 months was done by 2 trained psychologists using the Bayley Scales of Infant and Toddler Development, Third Edition (BSID-III), which measures the major areas of child development, including cognitive, language, motor, social-emotional, and adaptive functioning. In our study, inter-rater reliability was very high for the BSID-III cognitive score (intraclass correlation coefficient [ICC] 0.98, 95% CI: 0.97–0.99), language score (ICC 0.99, 95% CI: 0.99–1.00), and motor score (ICC 0.93, 95% CI: 0.90–0.97).

### Confounding variables

Selected PUFAs—linoleic acid (LA, 18:2 ω6), alpha-linoleic acid (ALA, 18:3 ω3), arachidonic acid (ARA, 20:4 ω6), eicosapentaenoic acid (EPA, 20:5 ω3), docosapentaenoic acid (DPA, 22:5 ω3), and docosahexaenoic acid (DHA, 22:6 ω3)—were measured in maternal serum collected during weeks 20 to 22 or week 32 of gestation. Concentrations of manganese (Mn), copper (Cu), zinc (Zn), arsenic (As), selenium (Se), cadmium (Cd), and lead (Pb) were measured in cord blood.

Maternal general intelligence was tested by using Raven’s Progressive Matrices, which consists of geometric-analogy–like visual problems that require the test-taker to choose, from a set of alternative answers, the correct missing figure to compete a matrix with a missing entry.

A detailed questionnaire was used to record additional information on socioeconomic status, maternal lifestyle, and pregnancy history. An update of this information—as well as information on breastfeeding, child intake of fish until age 18 months, and concurrent daycare attendance—was collected from the supplementary questionnaire.

### Chemical analysis

Hair samples were stored in Hg-free transparent plastic bags in a dark, uncontaminated area and were periodically sent to Jozef Stefan Institute (JSI) in Ljubljana for analysis. For each woman, one 1200-µL aliquot of blood, two 1500-µL aliquots and one 1000-µL aliquot of cord blood, and one 1200-µL aliquot of plasma from cord blood were stored in freezers (temperature, <−24°C) and then sent or transported in a frozen state (on dry ice) to the laboratories for analysis (namely, the JSI laboratory, for determination of THg and MeHg and for multi-elemental analysis, and the University of Ulster laboratory, for fatty acid determination).

A detailed description of the methods for the maternal measurements is provided in the Supplemental Materials. In short, THg in hair and cord blood was measured by thermal combustion at 650°C, amalgamation, and atomic absorption spectrometry using a direct mercury analyzer (Milestone, USA). MeHg in hair was measured by gas chromatography–electron capture detection (GC-ECD), whereas MeHg in cord blood was measured by a cold vapor atomic fluorescence detector (CVAFS). As, Cd, Cu, Mn, Pb, Se, and Zn in blood samples were measured by inductively coupled plasma–mass spectroscopy (ICP-MS). Total lipid was extracted from serum samples and then esterified. Fatty acid methyl esters were quantified using a gas chromatograph–mass spectrometer.

### Statistical analysis

Only data from children born during or after week 37 of gestation who had least 1 measure of Hg exposure and underwent BSID-III testing at 18 ± 2 months were included in the final analyses.

The main exposures of interest were maternal fish intake during pregnancy and THg concentration in biological samples from mothers and children. Fish intake during pregnancy was estimated from the detailed 138-item FFQ completed by the mothers soon after delivery. The questionnaire included 7 quantitative questions on fish, which addressed the frequency of consumption of 150-gram servings of fish, crustaceans, and mollusks (cooked according to different recipes), and fish in oil. The frequency distribution of those variables was described by using absolute numbers and percentages of subjects in each category. For each fish item, conversion from categories of consumption into continuous intakes of fish servings was done by assigning to each category a consumption level equal to the median value for that category (eg, 2–4 times/week became 3 times/week). Overall fish intake was calculated by summing the estimated weekly intake of all fish types.

Distributions of fish intake and Hg concentration were represented by arithmetic means and SDs, quartiles, and minimum-maximum ranges. Geometric means for Hg concentration were also calculated. Normality of distribution was assessed by the Shapiro-Wilk test. MeHg concentration in biological samples, which was known in a subsample of the cohort, was compared with THg concentration among the corresponding subjects.

The main outcomes of interest were the BSID-III composite cognitive, language, motor, social-emotional, and adaptive behavior scores. The distributions of scores were represented by arithmetic means and SDs, quartiles, and minimum-maximum ranges. Differences between groups were assessed by the Wilcoxon rank sum test (for continuous variables) and the χ^2^ test (for categorical variables). The associations of fish intake with concentrations of Hg and PUFAs were investigated by means of Spearman correlations. Crude associations between THg concentrations and each BSID-III score were analyzed using linear regression analyses. Separate models were built for each composite score. Because the distribution of THg in biological samples was not normal according to the Shapiro-Wilk test, THg was included in the regression models after logarithmic transformation. Models with no logarithmic transformation were also built for the purpose of sensitivity analysis. Other models were built with THg in maternal hair categorized into 4 groups (<500, 500–999, 1000–1999, and ≥2000 ng/g).

Multivariate linear regression was used to assess the association between Hg and neurodevelopment, after adjustment for potential confounding variables. The following covariates are hypothesized to affect child neurodevelopment and were thus considered in the analyses: maternal intake of fresh, frozen, and canned fish during pregnancy, concentrations of PUFAs (LA, ALA, ARA, EPA, DPA, and DHA) in maternal serum during pregnancy (included in the models either as individual concentrations, as percentages of the total concentration of fatty acids, as the sum of EPA, DPA, DHA, and ALA [ω-3] and the sum of ARA and LA [ω-6], or as the ratio of ω-6 to ω-3), the child’s sex, birth weight, gestational age, maternal intelligence quotient (IQ), maternal age at delivery, BMI before pregnancy, weight gain, marital status at delivery, socioeconomic (SES) index (adapted from Bennett et al^28^), size of the home, number of adults and children living in the home, cigarettes smoked and alcohol intake during pregnancy, breastfeeding history (any vs none, exclusiveness up to 4 months, duration up to 18 months), child intake of fish until age 18 months, daycare attendance at age 18 months, and time of BSID-III testing (AM vs PM). Models analyzing cord blood also included concentrations of Mn, Cu, Zn, As, Se, Cd, and Pb in cord blood as potential confounders.

Because the data for most covariates were derived from questionnaires, which can be incomplete, and to avoid loss of statistical power in multivariate analyses because of missing values, imputation was done for some variables, assuming that it was completely random. Among the covariates considered as potential confounders, only those associated with at least 1 BSID-III outcome (*P* < 0.10) were included in the final models.

We used analyses stratified by the child’s sex to assess whether the effect of Hg on neurodevelopment differed in boys and girls.

β coefficients are presented as estimates of effect, and *P*-values were used to assess statistical significance. Model *R*^2^ is presented as a measure of model fit.

## RESULTS

In total, 900 pregnant women were enrolled in the cohort; 767 (85%) of these women remained in the study at delivery, and 632 children underwent BSID-III testing at age 18 months (82% of those born within the cohort). The mothers of children lost to follow-up had a significantly lower SES index (median 2.50 vs 2.75, *P* = 0.0261) and IQ (median 118 vs 125, *P* = 0.0015), and were less likely to be married (85.5% vs 90.4%) and more likely to be separated (8.4% vs 3.3%, *P* = 0.0371), than those who were followed-up, whereas employment status and age at delivery (median 34 vs 33 years, *P* = 0.4374) were similar.

After excluding preterm births and 3 children who underwent BSID-III testing after age 20 months, data from 606 children were used in the final analyses. The sociodemographic characteristics of those children and their mothers are presented in Table 1.

**Table 1.** Sociodemographic characteristics of 606 Italian children and their mothers

Maternal age at delivery, years, mean ± SD (median)	33.3 ± 4.3 (33)
Maternal BMI before pregnancy, kg/m^2^, mean ± SD (median)	23.3 ± 14.5 (22.2)
Weight gain during pregnancy, kg, mean ± SD (median)	13.3 ± 4.2 (13)
Country of birth of mother, *n* (%)	
Italy	563 (92.9)
Other	43 (7.1)
Marital status of mother at delivery, *n* (%)	
Married/living with partner	541 (89.3)
Separated/divorced	20 (3.3)
Single	38 (6.3)
Not reported	7 (1.1)
Maternal educational level, *n* (%)	
Elementary school	5 (0.8)
Middle school	95 (15.7)
High school	293 (48.3)
University degree	211 (34.8)
Not reported	2 (0.3)
Maternal occupational status, *n* (%)	
Employed	508 (83.8)
Housewife	48 (7.9)
Seeking work	27 (4.4)
Student	6 (1.0)
Stopped working	8 (1.3)
Other/missing	9 (1.5)
Cigarettes smoked during pregnancy by mother, mean ± SD (median)	144 ± 560 (0)
Alcoholic drinks per week during pregnancy, mean ± SD (median)	1.6 ± 3.3 (0.3)
Raven’s Progressive Matrices score, mean ± SD (median)	119 ± 11 (125)
Socioeconomic index of family, mean ± SD (median)	2.6 ± 0.9 (2.7)
House ownership by family, *n* (%)	
Yes	469 (77.4)
No	130 (21.4)
Not reported	7 (1.1)
Home size	
<50 m^2^	41 (6.8)
50–100 m^2^	405 (66.8)
>100 m^2^	153 (25.2)
Not reported	7 (1.1)
Number of adults living in home (including the mother), *n* (%)	
1–2	568 (93.7)
>2	29 (4.8)
Not reported	9 (1.5)
Number of children living in home (including the newborn), *n* (%)	
1	339 (55.9)
2	200 (33.0)
3	39 (6.4)
>3	11 (1.8)
Not reported	17 (2.8)
Exposure to environmental tobacco smoke at home, *n* (%)	
Yes	120 (19.8)
No	486 (80.2)
Sex of child, *n* (%)	
Male	307 (50.7)
Female	299 (49.3)
Birth weight of child, grams, mean ± SD (median)	3419 ± 453 (3400)
Gestational age, weeks, *n* (%)	
37	33 (5.4)
38	94 (15.5)
39	163 (26.9)
40	166 (27.4)
41	119 (19.6)
42	31 (5.1)

Abbreviation: BMI, body mass index.

Mean maternal consumption of fish during pregnancy was 1.06 servings/week (SD 0.99, median 0.92, range 0–6), when only fresh or frozen fish were considered, and 1.69 servings/week (SD 1.30, median 1.38, range 0–7) when fresh, frozen, and canned fish were included. The mean intake of all fish, mollusks, and crustaceans was 2.33 servings/week (SD 1.71, median 1.84, range 0–11). The fish species that were consumed most frequently were tuna (mean intake: 0.64 times/week; mostly canned), sea bass (0.34 times/week; mostly fresh and of local origin), gilt-head bream (0.32 times/week; mostly fresh and of local origin), Mediterranean shad (0.21 times/week; mostly fresh and of local origin), cod (0.21 times/week; mostly frozen), trout/salmon (0.17 times/week; mostly fresh and of differing origin), plaice (0.16 times/week; mostly frozen), mackerel (0.14 times/week; mostly fresh and of local origin), and Dover sole (0.13 times/week; mostly fresh and of differing origin).

Table 2 shows the distributions of THg and MeHg concentrations in various biological samples, PUFAs in maternal serum, and BSID-III composite scores.

**Table 2.** Distribution of Hg concentrations (ng/g) in different biological samples, PUFAs in maternal serum in pregnancy (mg/ml), and BSID-III composite scores of children assessed at 18 months

	*n*	Arithmetic mean^a^	SD	25th percentile	Median	75th percentile	Minimum	Maximum
Mercury (ng/g)								
Maternal hair during pregnancy								
THg	604	1061	1028	481	788	1281	17	13 520
MeHg	220	1674	1046	1114	1382	1885	356	10 132
MeHg/THg (%)	220	0.89	0.16	0.84	0.99	1	0.16	1
Maternal blood during pregnancy								
THg	606	3.14	3.44	1.18	2.35	3.98	0.05	39.60
MeHg	236	4.46	4.04	2.39	3.46	5.30	0.37	39.60
MeHg/THg (%)	236	0.88	0.19	0.81	0.99	1	0.33	1
Maternal urine in pregnancy								
THg	589	1.11	1.51	0.29	0.71	1.40	0.01	18.61
Cord blood								
THg	457	5.54	4.83	2.40	3.97	7.02	0.12	32.76
MeHg	169	7.71	6.06	3.97	6.07	9.63	1.20	54.80
MeHg/THg (%)	167	0.87	0.13	0.78	0.90	1	0.36	1
Breast milk								
THg	492	0.33	1.31	0.11	0.18	0.28	0	28.30
MeHg	182	0.17	0.15	0.07	0.14	0.23	0.01	1.09
MeHg/THg (%)	182	0.58	0.29	0.31	0.60	0.84	0.01	1
PUFAs (mg/mL)								
ω-3								
18:3 ALA	589	0.023	0.007	0.018	0.022	0.026	0.016	0.062
20:5 EPA	589	0.039	0.035	0.024	0.026	0.034	0.018	0.200
22:5 DPA	589	0.047	0.007	0.048	0.048	0.048	0.018	0.120
22:6 DHA	589	0.045	0.029	0.018	0.036	0.064	0.014	0.152
Total ω-3	589	0.154	0.054	0.114	0.136	0.176	0.072	0.406
ω-6								
18:2 LA	587	0.436	0.164	0.370	0.452	0.530	0.016	0.882
20:4 ARA	589	0.114	0.053	0.078	0.116	0.154	0.018	0.304
Total ω-6	587	0.551	0.183	0.462	0.564	0.658	0.036	1.066
Total ω-6/total ω-3	587	3.81	1.48	2.83	3.81	4.75	0.32	9.64
BSID-III								
Cognitive score	604	106.2	8.3	100	105	110	75	130
Language score	604	97.7	8.7	91	97	103	47	121
Motor score	604	101.4	6.0	97	100	107	67	115
Social-emotional score	429	105.4	15.9	95	105	115	60	145
Adaptive behavior score	426	101.8	12.8	93	101	111	62	136

^a^Geometric means were 785 for THg in hair, 2.15 for THg in maternal blood, 0.64 for THg in urine, 3.88 for THg in cord blood, and 0.18 for THg in breast milk.

Abbreviations: PUFA, polyunsaturated fatty acid; ALA, α-linoleic acid; EPA, eicosapentaenoic acid; DPA, docosapentaenoic acid; DHA, docosahexaenoic acid; LA, linoleic acid; ARA, arachidonic acid; BSID, Bayley Scales of Infant and Toddler Development.

THg in maternal hair (*r* = 0.34, *P* < 0.0001), maternal blood (*r* = 0.33, *P* < 0.0001), and cord blood (*r* = 0.42, *P* < 0.0001) moderately correlated with reported intake of fish. Lower correlations were observed for THg in breast milk (*r* = 0.27, *P* < 0.0001) and maternal urine (*r* = 0.13, *P* = 0.0020). The fish species that most strongly correlated with THg, both in hair and in cord blood, were sea bass (*r* = 0.37 and *r* = 0.40, respectively), gilt-head bream (*r* = 0.34 and *r* = 0.37), and common pandora (*r* = 0.31 and *r* = 0.29) (all *P*-values <0.0001).

Reported intake of fish also weakly correlated with both serum concentration of DHA and with its percentage of total serum fatty acids (*r* = 0.08, *P* = 0.0569 and *r* = 0.09, *P* = 0.0298, respectively). No other significant correlations were found between maternal fish intake and maternal serum fatty acids during pregnancy.

Crude and adjusted associations between BSID-III composite scores and the logarithm of THg in maternal hair and cord blood are shown in Table 3. No significant inverse associations were observed between Hg in hair or cord blood and any of the BSID-III scores. Significant positive associations were found with language score, although these associations did not remain significant after covariate adjustment.

**Table 3.** Associations between BSID-III composite scores at age 18 months and the logarithm of THg concentration (ng/g) in biological samples

	Model 1^a^		Model 2^b^		Model 3^c^		Model 4^d^	
				
	β	*P*	β	*P*	β	*P*	β	*P*
**COGNITIVE**								
Ln [THg] maternal hair	0.4447	0.2945	0.3952	0.3816	0.3735	0.4015	−0.0022	0.9967
	*n* = 602		*n* = 602		*n* = 585		*n* = 505	
	*R*^2^ < 0.01		*R*^2^ < 0.02		*R*^2^ = 0.01		*R*^2^ = 0.08	
Ln [THg] cord blood	0.5198	0.2268	0.4577	0.3301	0.5898	0.1880	0.0518	0.9232
	*n* = 455		*n* = 455		*n* = 446		*n* = 378	
	*R*^2^ < 0.01		*R*^2^ < 0.01		*R*^2^ = 0.02		*R*^2^ = 0.10	
**LANGUAGE**								
Ln [THg] maternal hair	1.2389	0.0051	1.0551	0.0249	1.2995	0.0052	0.8464	0.1055
	*n* = 602		*n* = 602		*n* = 585		*n* = 505	
	*R*^2^ = 0.01		*R*^2^ = 0.01		*R*^2^ = 0.02		*R*^2^ = 0.17	
Ln [THg] cord blood	0.8298	0.0733	0.6359	0.2084	0.9484	0.0502	0.4142	0.4556
	*n* = 455		*n* = 455		*n* = 446		*n* = 378	
	*R*^2^ = 0.01		*R*^2^ = 0.01		*R*^2^ = 0.02		*R*^2^ = 0.19	
**MOTOR**								
Ln [THg] maternal hair	−0.2161	0.4852	−0.2895	0.3753	−0.3804	0.2351	−0.1875	0.6160
	*n* = 602		*n* = 602		*n* = 585		*n* = 505	
	*R*^2^ < 0.01		*R*^2^ < 0.01		*R*^2^ = 0.01		*R*^2^ = 0.08	
Ln [THg] cord blood	−0.0818	0.7926	−0.1815	0.5933	−0.0605	0.8527	0.1557	0.6844
	*n* = 455		*n* = 455		*n* = 446		*n* = 378	
	*R*^2^ < 0.01		*R*^2^ < 0.01		*R*^2^ = 0.01		*R*^2^ = 0.12	
**SOCIAL-EMOTIONAL**								
Ln [THg] maternal hair	1.5852	0.0867	0.9209	0.3493	1.800	0.0645	1.7727	0.1119
	*n* = 427		*n* = 427		*n* = 416		*n* = 362	
	*R*^2^ < 0.01		*R*^2^ = 0.01		*R*^2^ = 0.02		*R*^2^ = 0.15	
Ln [THg] cord blood	0.4978	0.6026	−0.5574	0.5976	0.4637	0.6484	−0.0711	0.9523
	*n* = 322		*n* = 322		*n* = 317		*n* = 271	
	*R*^2^ < 0.01		*R*^2^ = 0.02		*R*^2^ = 0.01		*R*^2^ = 0.19	
**ADAPTIVE BEHAVIOR**								
Ln [THg] maternal hair	1.4698	0.0646	0.9254	0.2708	1.5504	0.0586	0.5460	0.5556
	*n* = 424		*n* = 424		*n* = 414		*n* = 362	
	*R*^2^ = 0.01		*R*^2^ = 0.02		*R*^2^ = 0.02		*R*^2^ = 0.13	
Ln [THg] cord blood	0.5763	0.4840	−0.2051	0.8215	0.6007	0.4768	−0.5684	0.5659
	*n* = 317		*n* = 317		*n* = 313		*n* = 271	
	*R*^2^ < 0.01		*R*^2^ = 0.01		*R*^2^ = 0.02		*R*^2^ = 0.16	

^a^Crude association.

^b^Adjusted for maternal intake of fish in pregnancy.

^c^Adjusted for fatty acid concentration in maternal serum in pregnancy and for the proportion of 18:2 ω-6, 18:3 ω-3, 20:4 ω-6, 20:5 ω-3, 22:5 ω-3, and 22:6 ω-3 PUFAs.

^d^Adjusted for fish intake, fatty acids in maternal serum and proportion of PUFAs, sex, birth weight, maternal IQ, weight gain during pregnancy, marital status at delivery, SES index, number of children living in home, alcohol intake during pregnancy, breastfeeding history, child intake of fish until age 18 months, and daycare attendance at age 18 months.

Other measures of THg (maternal blood, urine, and breast milk) were not significantly associated with BSID-III cognitive, language, motor, or adaptive behavior scores (data not shown).

There were no significant associations between THg in maternal hair and any of the BSID-III scores, when THg concentration was categorized. When we used THg without logarithmic transformation, the direction and significance of associations with BSID-III scores were not altered. When we excluded from the analyses 3 records with a THg concentration in maternal hair greater than 6000 ng/g, the results changed only minimally (data not shown).

Using the cohort subsample with available MeHg data, univariate analyses revealed significant positive associations of MeHg in hair and cord blood with BSID-III cognitive scores (β = 2.6685, *P* = 0.0287 and β = 2.3170, *P* = 0.0135, respectively); however, these associations did not remain significant after adjustment for confounders (data not shown).

Analyses stratified by the child’s sex showed only 1 significant association: the logarithm of THg in maternal hair was associated with BSID-III language score among girls (β = 1.5291, *P* = 0.0445). No association was evident among boys (β = 0.3551, *P* = 0.6278).

Regarding the possible effect of maternal fish intake during pregnancy (reported servings/week) on child neurodevelopment, univariate models showed significant positive associations with BSID-III composite language (β = 0.5426, *P* = 0.0447), social-emotional (β = 1.4589, *P* = 0.0134), and adaptive behavior scores (β = 1.1384, *P* = 0.0150), although the association with language score was not significant in the fully adjusted models. However, positive associations remained between maternal fish intake and social-emotional scores in the model with THg as measured in cord blood (β = 1.8425, *P* = 0.0284).

In univariate models, no associations were found between BSID-III scores and maternal serum fatty acids, except for a positive association between ALA percentage and cognitive score (β = 1.6551, *P* = 0.0681), the direction and magnitude of which were unchanged in the fully adjusted models (β = 1.4084, *P* = 0.1685 for the model with THg in hair, and β = 1.6232, *P* = 0.1186 for the model with THg in cord blood). In the fully adjusted models, an analogous association was present with language score (β = 1.5894, *P* = 0.1193 and β = 1.4689, *P* = 0.2288, respectively). Table 4 shows that when absolute concentrations of PUFAs were included in the models, none of the associations was statistically significant. An inverse association was found between the ratio of total ω-6 to total ω-3 fatty acids and language score (*P* = 0.0707). No significant differences were noted between boys and girls in analyses stratified by the child’s sex.

**Table 4.** Associations between BSID-III composite scores at age 18 months and PUFA concentrations in maternal serum (mg/ml) during pregnancy

	BSID-III composite cognitive score			BSID-III composite language score			BSID-III composite motor score			BSID-III composite social-emotional score			BSID-III composite adaptive behavior score		
					
	β (*P*)^a^	β (*P*)^b^	β (*P*)^c^	β (*P*)^a^	β (*P*)^b^	β (*P*)^c^	β (*P*)^a^	β (*P*)^b^	β (*P*)^c^	β (*P*)^a^	β (*P*)^b^	β (*P*)^c^	β (*P*)^a^	β (*P*)^b^	β (*P*)^c^
ω-3															
18:3 ALA	56.8287 (0.3533)	—	—	76.7142 (0.2095)	—	—	−25.6522 (0.5565)	—	—	22.3358 (0.8719)	—	—	−99.1767 (0.3547)	—	—
20:5 EPA	0.0734 (0.9946)	—	—	8.9996 (0.4064)	—	—	−7.9093 (0.3069)	—	—	−0.2595 (0.9917)	—	—	17.5969 (0.3781)	—	—
22:5 DPA	−58.2675 (0.2780)	—	—	−19.5536 (0.7152)	—	—	−46.7118 (0.2224)	—	—	−44.9412 (0.1820)	—	—	−36.2873 (0.6129)	—	—
22:6 DHA	10.6137 (0.4935)	—	—	12.1836 (0.4310)	—	—	7.2911 (0.5092)	—	—	−45.4528 (0.1820)	—	—	31.0207 (0.2562)	—	—
Total ω-3	—	3.0549 (0.6607)	—	—	10.1456 (0.1441)	—	—	−4.1162 (0.4064)	—	—	−18.1834 (0.2460)	—	—	13.0968 (0.3022)	—
ω-6															
18:2 LA	0.9615 (0.6896)	—	—	−3.2557 (0.1759)	—	—	0.9968 (0.5613)	—	—	−6.9937 (0.2053)	—	—	−1.4397 (0.7427)	—	—
20:4 ARA	0.5338 (0.9497)	—	—	−5.7276 (0.2095)	—	—	1.5919 (0.7917)	—	—	3.0915 (0.8708)	—	—	−16.2127 (0.2914)	—	—
Total ω-6	—	1.6888 (0.4245)	—	—	−2.8919 (0.1705)	—	—	1.3348 (0.3755)	—	—	−7.2464 (0.1278)	—	—	−3.7132 (0.3288)	—
Total ω-6 /ω-3	—	—	0.0574 (0.8174)	—	—	−0.4481 (0.0707)	—	—	0.0892 (0.6143)	—	—	−0.3306 (0.5492)	—	—	−0.5027 (0.1698)

^a^Linear regression model including concentrations of ALA, EPA, DPA, DHA, LA, and ARA (mg/ml), the logarithm of THg concentration in maternal hair during pregnancy, maternal fish intake, the child’s sex and birth weight, maternal IQ, weight gain during pregnancy, marital status at delivery, SES index, number of children living in home, alcohol intake during pregnancy, breastfeeding history, child intake of fish until age 18 months, daycare attendance at age 18 months.

^b^Linear regression model including concentrations of ω-3 and ω-6 PUFAs, plus the covariates listed above.

^c^Linear regression model including ω-6/ω-3 ratio, plus the covariates listed above.

Abbreviations: PUFA, polyunsaturated fatty acid; ALA, α-linoleic acid; EPA, eicosapentaenoic acid; DPA, docosapentaenoic acid; DHA, docosahexaenoic acid; LA, linoleic acid; ARA, arachidonic acid; BSID, Bayley Scales of Infant and Toddler Development.

In our multivariate models, maternal IQ was consistently associated with the child’s cognitive, language, motor, and social-emotional scores (β = 0.0710, 0.0696, 0.0524, and 0.1891, respectively; *P*-values = 0.0527, 0.0569, 0.0450, and 0.0219, respectively). No significant association was found with adaptive behavior score (β = −0.0082, *P* = 0.9010). Girls performed better than boys on the cognitive (β = 1.3999, *P* = 0.0628), language (β = 4.7544, *P* < 0.0001), social-emotional (β = 6.8962, *P* < 0.0001), and adaptive behavior tests (β = 5.4241, *P* < 0.0001), whereas the difference in motor scores was not significant (β = 0.7158, *P* = 0.1819). Duration of fresh fish intake in children was positively associated with cognitive scores (β = 0.2002, *P* = 0.0346) and with the adaptive behavior score (β = 0.5041, *P* = 0.0037). No associations were found with language (β = 0.0944, *P* = 0.3168), motor development (β = −0.0007, *P* = 0.9913), or social-emotional score (β = 0.3281, *P* = 0.1327).

## DISCUSSION

In this Italian cohort, maternal fish intake during pregnancy was moderate (on average, 1.69 servings/week)—much lower than consumption reported in the Seychelles (12 fish meals a week)^29^ but comparable to that of a US cohort of pregnant women.^30^ Hg concentrations in biological samples from mothers and children were consistently low (on average 1000 ng/g in maternal hair; only 10 subjects had a THg concentration >4000 ng/g and only 3 subjects had a concentration >6000 ng/g). In the spectrum of relatively low concentrations that we measured, there was no evidence that prenatal Hg exposure had an adverse effect on child neurodevelopment. In fact, regardless of the biological sample considered for determination of THg concentration, all the associations of THg with BSID-III composite scores were non-significant after adjustment for a number of potential confounders. The consistency of the results obtained with different biological samples indicates that the present findings do not depend on the particular biological matrix chosen to ascertain exposure.

In the Seychelles Child Development Study, a possible explanation for the inconsistent pattern of associations between MeHg and child neurodevelopment was residual confounding owing to unmeasured covariates, most likely nutritional factors also present in fish.^29^ However, we found very little change from the crude estimates of the effect of THg on BSID-III scores to those adjusted for reported maternal fish intake or serum fatty acid concentrations during pregnancy, which suggests that in this population with a limited fish intake the effects of Hg were not obscured by nutritional status.

In the fully adjusted models, we found no effect of reported maternal fish intake on child neurodevelopment and very little correlation of such intake with serum concentrations of LCPUFAs. Possible reasons for these findings regarding fish and LCPUFAs are that fish intake was misreported on the FFQ, that there were errors in estimating weekly servings based on FFQ consumption categories, that fish marketed in the study area were low in LCPUFAs and other beneficial nutrients, that the intake of fish in our cohort was so low that fish was not the predominant source of LCPUFAs, and that the most important PUFAs were preferentially transferred from the placenta to the fetus during the last 10 weeks of pregnancy, which could cause the distribution of PUFAs in blood to differ from that in the food sources.^31^ Further research, and measurement of LCPUFA concentrations in fish marketed around Trieste, will help clarify this issue.

Contrary to our expectations, LCPUFAs had little effect on child neurodevelopment. Associations were only observed for ALA (direct association) and for ω-6/ω-3 ratio (inverse association) and language scores, although they were not significant. In the Seychelles Child Development Nutrition Study, an inverse association was found between ω-6/ω-3 ratio and the psychomotor development index of the BSID-II. In that study, no association was found between LCPUFAs and the mental development index of the BSID-II, whereas a positive association was found between the psychomotor development index and ω-3 LCPUFAs at ages 9 months and 30 months.^20^^,^^21^ In our study, we assessed neurodevelopmental outcomes at age 18 months; however, it is possible that the beneficial effects of LCPUFAs transferred from mother to fetus via the placenta are transient and that other sources of LCPUFAs become important as the child grows. This possibility could explain why we found little effect. There is inconclusive evidence from randomized controlled clinical trials that LCPUFA supplementation of formula milk or supplementation of breastfeeding mothers improves neurodevelopmental outcomes of infants born at term.^32^^,^^33^ In our cohort, however, the duration of child intake of fish was more predictive of BSID-III cognitive score at age 18 months than were nutrients received during the intrauterine period.

Another possible reason for the lack of association between LCPUFAs in maternal serum and child neurodevelopment is that, in the last weeks of pregnancy (when most fat deposition occurs in the fetus), the delivery of individual fatty acids to the fetus tends to be more balanced than the fatty acid composition of the maternal blood, due to the buffering capacity of maternal fat stores and the regulating effect of the placenta, ie, the placenta preferentially transfers the most important PUFAs to the fetus.^31^

Among the numerous additional variables that we evaluated in this study, only a few were associated with BSID-III scores. As expected, maternal IQ consistently influenced cognitive, language, and motor outcomes. Girls had better language performance, as in other studies.^34^^,^^35^ Child intake of fresh fish also appeared beneficial for cognitive development.

Despite the inclusion of a large number of factors in the models, we were only able to explain a small proportion of the variability, as indicated by the small model *R*^2^, which indicates that child neurodevelopment in our society is a multifactorial phenomenon, influenced by many variables, and that residual confounding may exist in our effect estimates. In addition, our study was limited to a single neurodevelopmental assessment, at 18 age months. Further testing at different child ages could yield different results and should also consider indicators of the home environment and stimuli received by the child within the family context.^36^^,^^37^

Regarding other potential confounders, we measured prenatal exposures to a number of metals and elements other than Hg, but we had no data on polychlorinated biphenyl (PCB) exposure, which is also derived from fish and other foods. In a recent article, Miklavcic et al^38^ described PCB levels in fish marketed in neighboring Slovenia and, as compared with the tolerance limits recommended by the US Food and Drug Administration, those levels did not represent a health risk for humans. Measurement of PCBs in samples of fish marketed in Italy or in biological samples of a subset of our cohort could help determine if PCB exposure was an issue in this population.

Another limitation of this study is that, for budget reasons, MeHg data were limited to a subset of the cohort (approximately one-third of subjects, who were selected from among subjects with the highest THg hair levels). MeHg is the organic form of Hg, which accumulates in fish and enters the human body through the food chain. Therefore, this measure is the most appropriate exposure for study in a general population that has a diet including fish and without obvious substantial occupational or environmental exposure to inorganic Hg, as in our population. In our cohort, analyses of associations between BSID-III scores and MeHg were performed on a restricted group of subjects, who were not necessarily representative of the whole cohort. However, the present results were consistent with those obtained using THg in the entire cohort and showed no adverse effect of Hg on neurodevelopment. In addition, among subjects with MeHg data, the ratio of MeHg to THg was very high (median, 0.99 in hair and 0.90 in cord blood). Therefore, we assumed that in the majority of cohort members most Hg was methylated and that THg could be used as a proxy for MeHg.

Finally, our study identified factors that influence child neurodevelopment, eg, maternal IQ, but may not have had sufficient statistical power to detect the effects of Hg if they were very subtle. Our northern Italian cohort is part of a larger multi-country cohort study and power calculations were based on the pooled analysis of data from all the participant countries.^24^

### Conclusions

Prenatal Hg exposure in our cohort was low, and we detected no adverse neurodevelopmental effects at these levels. In addition, maternal fish intake and maternal serum LCPUFAs had little effect on child neurodevelopment. In this child population, performance on the BSID-III was influenced by child intake of fish, maternal IQ, and the child’s sex.

## ONLINE ONLY MATERIALS

eMaterial. Chemical analysis.
