# Supplementary material for: Associations of Prenatal Mercury Exposure From Maternal Fish Consumption and Polyunsaturated Fatty Acids With Child Neurodevelopment: A Prospective Cohort Study in Italy
Source: J Epidemiol. 2013 Sep 5;23(5):360–70. doi: 10.2188/jea.JE20120168 (PMC3775530; doi:10.2188/jea.JE20120168)
Supplement: eMaterial. — Chemical analysis. [file je-23-360-s001.pdf]

eMaterial

Chemical analysis

Mercury

Determination of THg in hair and cord blood

THg in hair and cord blood were determined by thermal combustion at 650°C, amalgamation, and atomic absorption spectrometry using a direct mercury analyzer (Milestone, USA). The procedure has been described in detail elsewhere.[1] About 0.020 g of human hair or about 0.200 g of cord blood was weighed in a sample boat. The reference material NIES 13 (human hair) was used to check the accuracy of the results of THg in hair, and the resulting value was in good agreement with the reference value. The reference material Seronorm Trace Elements in Whole Blood L-1 was used to check the accuracy of the results for THg in cord blood, and the resulting value was in good agreement with the reference value. The limit of detection (LOD) of the method—defined as 3 times the SD of the blank sample) was 0.2 ng/g for a hair sample and 0.02 ng/g for a cord blood sample, while the limit of quantitation (LOQ) was 0.7 ng/g for a hair sample and 0.07 ng/g for a cord blood sample. Although the sample mass was low, the estimated precision of determination of THg in hair samples was 7% (k=2, 95% CI). The estimated precision for determination of THg in blood samples at levels greater than or equal to 1 ng/g was 7% (k=2, 95% CI),

while at lower levels (<1 ng/g) it was 14% (k=2, 95% CI). In addition, the stability of the blood samples during freezing and defrosting was checked at 3 different THg concentration ranges. Samples were defrosted for 2 hours at room temperature, and freezing and defrosting were repeated 3 times. In sets of blood samples in which THg in hair was less than 1100 ng/g, the THg level in blood decreased by up to 20%, while no decrease in concentration was observed for the higher THg concentration range in blood.

Determination of MeHg in hair

About 0.015 to 0.030 g of a hair sample was weighed into a Teflon tube, 10 ml of 6M of HCl (Suprapur; Merck, Germany) was added, and the mixture was shaken overnight. MeHg in the extract was subsequently back-extracted with 0.5 ml of toluene. Two microliters of the toluene was injected into a packed gas chromatography column, and MeHg was measured by gas chromatography–electron capture detection (GC-ECD). A complete description of the method has been published elsewhere.[2,3] The reference material NIES 13 (human hair) was used to check the accuracy of the results for MeHg in hair, and the resulting value was in good agreement with the reference value. The estimated precision of the method (calculated using the SD of MeHg determined in the reference material) was 12% (k=2, 95% CI). The LOD of the method (calculated on the

basis of 3 SDs) was 0.2 ng/g in the hair sample, and the LOQ was estimated at 0.7 ng/g in the hair sample.

Determination of MeHg in cord blood

About 200 mg of cord blood was weighed directly in a 30-ml screw-capped Teflon vial to which 6 ml of a mixture of 5% H2SO4 (Suprapur; Merck, Germany), 18% KBr (Merck, Germany, pa), and 1.0 ml of a 1 M solution of CuSO4 (Merck, Germany, pa) was added. After vigorously shaking the vials for 15 minutes, 10 ml of CH2Cl2 (Suprasolv; Merck, Germany,) was added to each vial, and the vials were again shaken for 15 minutes and centrifuged for 5 minutes at 3200 rpm. The organic phase was separated from the aqueous phase in a Teflon separating funnel and collected in a 60-ml Teflon vial. An additional 5 ml of CH2Cl2 was added to the vials, and the same extraction was then repeated. Aqueous phase ethylation was performed by adding approximately 30 ml of Mili-Q water, evaporating the samples on a water bath at about 90°C, removing the remaining CH2Cl2 with nitrogen gas, adjusting the pH to 4.6 with acetate buffer (CH3COOH: Merck, Germany, Suprapur; KH2COOH: Merck, Germany, extra pure) in a Teflon reaction vial, and adding 50 µl of 1% NaBEt4 (Stream Chemicals, USA). The mixture was left at room temperature for 15 minutes and then ethylated MeHg as ethylmercury was purged onto a Tenax trap for 15 minutes with nitrogen gas. The Tenax trap was then connected to a flow of argon

and MeHg was thermally desorbed (180°C) onto an isothermal gas chromatographic (GC) column. Hg species were converted to Hg0 by pyrolysis at 600°C and measured by a cold vapor atomic fluorescence detector (CVAFS). The procedure has been described in detail elsewhere.[4,5] The accuracy of the results was checked by analyzing the standard reference materials Spiked Skim Milk Powder BCR 150, Non-Fat Milk Powder NIST 1549, and Seronorm Trace Elements Whole Blood L-1. The measured values were in good agreement with the certified and reference values. The estimated precision of MeHg determined in cord blood samples was 12% (k=2, 95% CI) at levels greater than or equal to 1 ng/g and 14% (k=2, 95% CI) at lower levels (<1 ng/g). The LOD of the method for MeHg determination in cord blood (calculated on the basis of 3 SDs) was 0.02 ng/g, while the LOQ was estimated at 0.05 ng/g for cord blood samples.

Elements

Determination of As, Cd, Cu, Mn, Pb, Se, and Zn in cord blood samples by inductively coupled plasma–mass spectroscopy (ICP-MS)

An aliquot of 0.3 mL of blood sample was diluted 10 times with an alkaline solution containing Triton X-100 and ethylenediaminetetraacetic acid disodium salt dehydrate (EDTA).[6] An internal standard solution containing Ga, Gd, Y, and Sc was added. For calibration, the standard addition procedure was performed. Measurements of prepared solutions were done with an Octapole Reaction

System (ORS) inductively coupled plasma–mass spectrometer (7500ce, Agilent) equipped with an ASX-510 Autosampler (Cetac) (instrumental conditions: Babington nebulizer, Scott-type spray chamber, spray chamber temperature 5°C, plasma gas flow rate 15 L/min, carrier gas flow rate 0.8 L/min, make-up gas flow rate 0.1 L/min, sample solution uptake flow rate 1 mL/min, RF power 1500 W, reaction cell gas helium 4 mL/min, isotopes monitored ^{55}Mn, ^{63}Cu, ^{66}Zn, ^{75}As, ^{77}Se, ^{78}Se, ^{111}Cd, ^{114}Cd, ^{206}Pb, ^{207}Pb, ^{208}Pb). The device was calibrated daily using a solution containing Li, Mg, Y, Ce, Tl, and Co. Quantification of all isotopes was performed using 1 central point of the spectral peaks and 3 repetitions. The reference material Seronorm Trace Elements Whole Blood L-1 (Sero) was used to check the accuracy of the results, and the resulting values were in good agreement with the reference values. The LODs for Cd, Pb, As, Se, Cu, Zn, and Mn (calculated as 3 times the SD of the blank sample) were 0.12, 1.3, 0.13, 5, 11, 20, and 1.7 ng/g, respectively, in blood samples.

Fatty acids

Total lipid was extracted from serum tissue using a method adapted from Folch et al.[7] The internal standard, heptadecanoic acid (C17:0), was added to all samples before extraction at a concentration of 1 mg/ml. The lipid extracts were esterified with boron trifluoride in methanol (Sigma Aldrich Company Limited). Fatty acid methyl esters were quantified using an Agilent 5975C GC MS device

(Agilent Technologies UK Limited, Stockport, UK) operated in split mode, with a BPX70 capillary GC column (SGE Analytical Science; length 60 mm, internal diameter 0.25 mm, and film thickness 0.25 mm), using He as the carrier gas. The samples were injected in split injection mode (50:1 ratio) at a temperature of 160°C, and the temperature was ramped at 2°C/min to 208°C (maintained for 10 minutes) and then at 2°C/min to 220°C (maintained for 10 minutes). Fatty acids were identified by their retention times as compared with those of commercially available fatty acid standards (Sigma Aldrich Company Limited) and were quantified by use of the internal standard heptadecanoic acid (C17:0) (Sigma Aldrich Company Limited), with reference to the standard curve.

References

1. EPA, 1998. Method 7473. Mercury in solids and solutions by thermal decomposition amalgamation and atomic absorption spectrometry.
2. Horvat M, May K, Stoepple M, Byrne AR. Comparative studies of methylmercury in biological and environmental samples. Appl Organomet Chem. 1988;2:515-524.
3. Horvat M, Byrne AR, May K. A modified method for the determination of methylmercury by gas chromatography. Talanta. 1990;37:207-212.
4. Horvat M, Liang L, Bloom NS. Comparison of Distillation with other current isolation methods for the determination of methyl mercury compounds in low level environmental samples, Part 2: Water. Anal Chim Acta. 1993;282:153-168.
5. Liang L, Horvat M, Bloom NS. An improved method for speciation of mercury by aqueous phase ethylation, room temperature precollection, GC separation and CV AFS detection. Talanta. 1994;3:371-379.
6. Barany E, Bergdal IA, Schutz A, Skerfving S, Oskarsson A. Inductively Coupled Plasma Mass Spectrometry for Direct Multi-element Analysis of Diluted Human Blood and Serum. JAAS. 1997;12:1005-1009.
7. Folch J, Lees M, Sloane Stanley GH. A simple method for the isolation and purification of total lipides from animal tissues. J Biol Chem. 1957;226:497-509.
